# APTw combined with mDixon−Quant imaging to distinguish the differentiation degree of cervical squamous carcinoma

**DOI:** 10.3389/fonc.2023.1105867

**Published:** 2023-01-25

**Authors:** Xing Meng, Shifeng Tian, Changjun Ma, Liangjie Lin, Xiaoxiao Zhang, Jiazheng Wang, Qingwei Song, Ai Lian Liu

**Affiliations:** ^1^ First Affiliated Hospital, Dalian Medical University, Dalian, Liaoning, China; ^2^ Radiology Department, Dalian Women and Children’s Medical Group, Dalian, Liaoning, China; ^3^ Radiology Department, Philips (China), Beijing, China

**Keywords:** magnetic resonance imaging, amide proton transfer weighted, modified Dixon-Quant, transverse relaxation rate, cervical squamous carcinoma

## Abstract

**Background:**

To investigate the value of amide proton transfer weighted (APTw) imaging combined with modified Dixon fat quantification (mDixon-Quant) imaging in determining the degree of differentiation of cervical squamous carcinoma (CSC) against histopathologic.

**Methods:**

Magnetic resonance imaging (MRI) data were collected from 52 CSC patients. According to histopathologic results, patients were divided into the poorly differentiated group (37 cases) and the well/moderately differentiated group (15 cases). The APTw value by APTw imaging and the fat fraction (FF) and transverse relaxation rate R2* values by mDixon-Quant were independently measured by two radiologists. Intra-class correlation coefficients (ICCs) were used to test the consistency of APTw, FF, and R2* values measured by the two observers. The Mann-Whitney U test was used to analyze the difference in each parameter between the two groups. Logistic regression analysis was used to assess the association between the degree of differentiation on histopathology and imaging parameters by APTw and mDixon Quant. The ROC curve was used to evaluate the diagnostic efficacy of various parameters and their combination in distinguishing the degree of CSC differentiation on histopathology. The DeLong test was used to access the differences among the area under the ROC curves (AUCs). The Pearson correlation coefficient was used to evaluate the correlation between APTw and mDixon-Quant imaging parameters.

**Results:**

The APTw means were 2.95 ± 0.78% and 2.05 (1.85, 2.65)% in the poorly and well/moderately differentiated groups, respectively. The R2* values were 26.62 (21.99, 33.31)/s and 22.93 ± 6.09/s in the poorly and well/moderately differentiated groups, respectively (P < 0.05). The AUCs of APTw, R2* , and their combination were 0.762, 0.686, and 0.843, respectively. The Delong test suggested statistical significance between R2* and the combination of APTw and R2* . R2* values showed a significant correlation with APTw values in the poorly differentiated group.

**Conclusions:**

APTw combined with mDixon-Quant can be used to efficiently distinguish the differention degrees of CSC diagnosed on histopathology.

## 1 Introduction

Cervical cancer is a malignant tumor that threatens the health and life of women worldwide. According to the global cancer data in 2018, cervical cancer ranks fourth in the incidence and mortality of female patients with malignant tumors (1). Cervical squamous carcinoma (CSC) is a common pathologic type of cervical tumors. The degree of tumor differentiation is a prognostic factor of CSC, especially during the early stages of the disease. The higher the pathologic tumor grade, the worse the degree of differentiation, the easier for the tumor to locally invade and form distant metastases, the higher the recurrence rate, the worse the prognosis, and the lower the survival rate (2–5). Histopathologic tumor types, the degree of tumor differentiation, and the stages of cervical cancer are key factors when choosing a treatment plan and assessing prognosis. Therefore, accurately determining the degree of cervical cancer differentiation on histopathology is critical.

Cervical biopsy is a common diagnostic method for cervical cancer, but it is an invasive method and not suitable for patients with cervical or vaginal stenosis. Besides, the accuracy of cervical biopsy can be affected by human factors, such as sample size, location of sampling, etc. MRI, as a noninvasive examination method, plays an important role in evaluation of the staging and degree of differentiation of cervical cancer. The modified Dixon fat quantification (mDixon-Quant) technique is a water-fat separation technology that enables semutaneous mapping of proton density fat fraction and transverse relaxation rate R2* = 1/ T2* (6), thus reflecting the fat contents and iron deposition in tissues. mDixon-Quant has been used to diagnose spinal lesions (7), detect liver lesions (8, 9), measure testicular and epididymal fat content (10), and quantitatively analyze muscle fat content (11).

Amide proton transfer weighted (APTw) imaging is a subtype of chemical exchange saturation transfer imaging, which use off-resonance saturation pulses to detect free protein and polypeptide molecules in cell cytoplasm without using an exogenous contrast agent (12). It has been widely explored for clinical applications, such as evaluating glioma grades (13), diagnosing and estimating the severity of Parkinson’s disease (14), differentiating benign and malignant tumors of the head and neck (15) and breast (16), diagnosing prostate cancer and performing risk assessments (17), evaluating the prognostic factors of rectal adenocarcinoma (18), and predicting the histologic grade of hepatocellular carcinoma (19).

To the best of our knowledge, the use of quantitative APTw and mDixon-Quant imaging to evaluate the degree of CSC differention compared with histopathology has not been reported. This study explored the value of APTw and mDixon-Quant in evaluating the degree of CSC differention and thus provides valuable information for preoperative diagnoses and treatments.

## 2 Materials and methods

### 2.1 Study population

The patients with cervical cancer who underwent MRI examinations from May 2019 to February 2022 were retrospectively identified from the database. We included patients who meet the criteria of 1) a tumor size > 1 cm in diameter; 2) had not received radiotherapy, chemotherapy, or any other treatment. Pelvic 3.0T MRI examinations were performed before the surgeries. The exclusion criteria were 1) incomplete pathologic information or lack of grading-related information, and 2) poor image quality. Finally, 52 cases of CSC were included in this study, which was divided into a poorly differentiated group (37 cases) and a well/moderately differentiated group (15 cases) based on the pathologic results. The CSC patients in current study were commonly associated with abnormal vaginal bleeding, while the clinical manifestations in patients with poorly differentiated and well/moderately tumors were similar (**Table 1**).

**Table 1:** Comparison of clinical data of patients in the poorly differentiated and well/moderately differentiated groups.

		Poorly differentiated group (n=37)	Well/moderately differentiated group (n=15)	t/χ^2^ values	P-value
Age (yr)		53.9 ± 11.1	55.2 ± 7.0	-0.488^*^	0.629
Menopause status	pre	14 (37.8%)	4 (26.7%)	0.588	0.443
	post	23 (62.2%)	11 (73.3%)		
Vaginal bleeding	with	27 (73.0%)	10 (66.7%)	0.207	0.907
	without	10 (27.0%)	5 (33.3%)		

*: t values.

### 2.2 Imaging protocol

MR scans, including T_2_ weighted imaging (T_2_WI), APTw, and mDixon-Quant, were performed on a 3.0T MRI scanner (Ingenia CX, Philips Healthcare, Best, the Netherlands) equipped with a 32-channel abdominal coil. MR scan parameters are listed in **Table 2**.

**Table 2:** T_2_WI, APTw, and mDixon-Quant scan parameters.

	TA (min/sec)	NSA	TR (ms)	FOV (mm^2^)	Pixel size (mm^2^)	Slice thickness/gap (mm)
T_2_WI	59s	1	3672/95	240×240	0.7×0.7	4.0/1.0
APTw	4 min 45s	1	5174/7.8	130×130	2.0×2.0	7.0/0.0
mDixon-Quant	15s	1	6	375×375	2.3×1.8	5.0/-2.5

TA, acquisition time; NSA, number of signal averages; TR, repetition time; FOV, field of view; T_2_WI, T2 weighted imaging; APTw, amide proton transfer weighted.

APTw imaging used a 3D fast spin-echo sequence. Signals were acquired with the radiofrequency saturation pulses applied at frequencies of ±2.7, ± 3.5, and ±4.3 ppm using the B1 intensity (rms) of 2 μT and a duration of 2 seconds for fitting of the Z-spectrum (with the water frequency definited as 0 ppm). The reference scan is obtained by setting the saturation frequency to -1540 ppm. The data from three different echo times were collected at the saturation frequency of +3.5 ppm to generate the B0 map for the B0 correction of the Z-spectrum in each image voxel. The APTw value was obtained by calculating the asymmetry of the traditional magnetization transfer effect at 3.5 ppm on both sides of the water signal (20).

mDixon-Quant uses a 3D gradient echo sequence. In this study, it measured images at the 6 echo times (TEs) of 1.11, 1.81, 2.61, 3.41, 4.21, and 5.01 ms, respectively. The post-processing of both APTw and mDixon-Quant imaging was performed on the MR console after data collection. After phase correction, accurate fat quantification was achieved with a seven-peak spectral fat model that enabled T2^*^ corrections (21). The proton density fat fraction (FF) map was computed as the ratio of the fat signal over the sum of fat and water signals.

### 2.3 Image processing and data analysis

**Figure 1:**
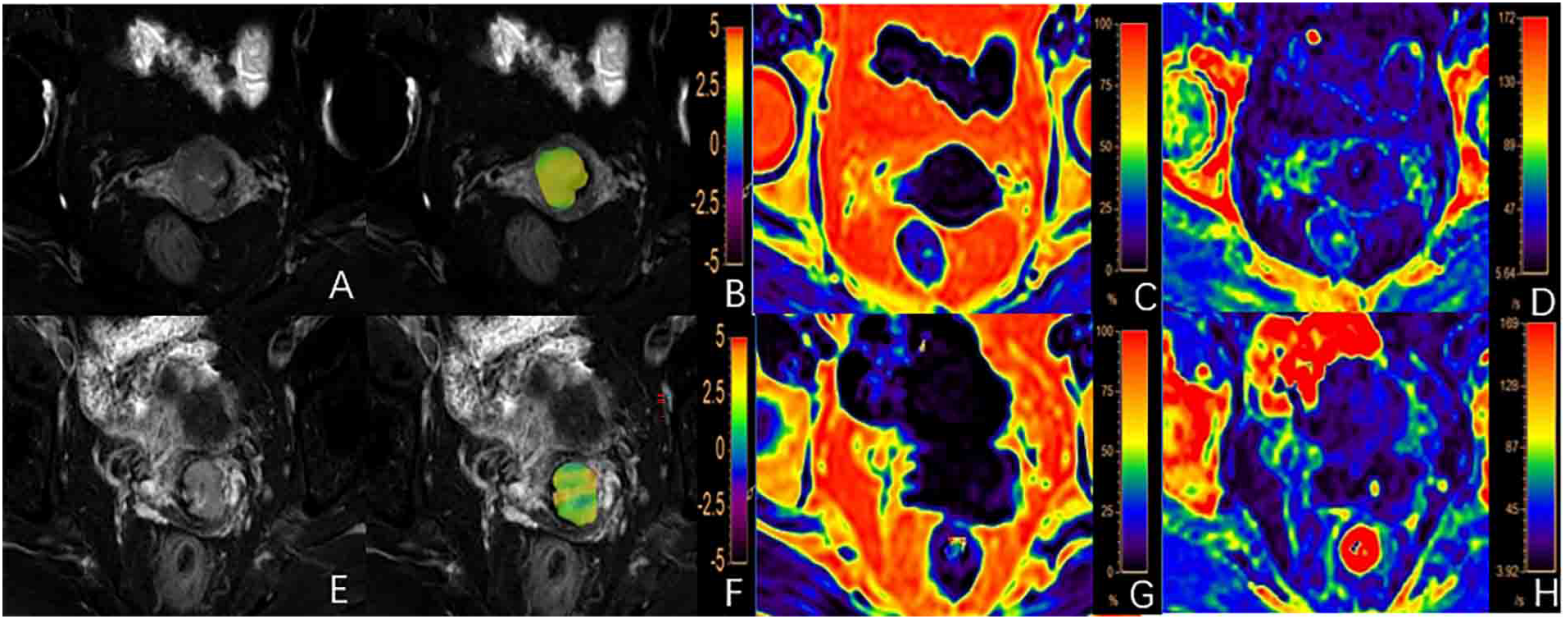
**(A-D)** A 62-year-old patient with a poorly differentiated cervical squamous carcinoma (CSC): **(A)** T_2_WI image, **(B)** fused APTw and T_2_WI image with the APTw value for the lesion of 2.45%; **(C, D)** FF and ${\text{R}}_{2}^{*}$ images with FF and ${\text{R}}_{2}^{*}$ values for the lesion of 2.69% and 19.62/s, respectively; **(E-H)** A 56-year-old patient with well/moderately differentiated CSC: **(E)** T_2_WI image; **(F)** fused APTw and T_2_WI image with an APTw value for the lesion of 2.05%; **(G, H)** The FF and ${\text{R}}_{2}^{*}$ images with FF and ${\text{R}}_{2}^{*}$ values for the lesion of 1.04% and 19.51/s, respectively.

Images were uploaded to the IntelliSpace Portal (ISP v9.0, Philips Healthcare) workstation for quantitative measurements. Regions of interest (ROIs) were drawn independently by two radiologists (both with 10 years of diagnostic experience in abdominal radiology), who were blinded to the clinical information and histopathologic results. The ROIs of lesions on the APTw images were delineated on the slice showing the largest lesion diameter with reference to the T_2_WI images (**Figure 1**). The ROIs aimed to encompass the entire tumor, excluding regions such as cystic changes, hemorrhage, necrotic areas, and tumor boundaries to avoid partial volume effects. ROIs of the mDixon-Quant images were drawn to match as closely as possible the position of the lesion drawn on the APTw images.

### 2.4 Statistical analysis

All statistical analyses were conducted with SPSS 26.0 software unless otherwise specified. Intra-class correlation coefficients (ICCs) were used to test the consistency of the APTw, FF, and R2* values determined by the two observers. The ICC < 0.40; 0.40 ≤ ICC < 0.75; and ICC ≥0.75 were regarded as poor, medium, and good consistency, respectively. Mean values by the two observers were used for subsequent statistical analysis. Continuous variables were compared between the poorly differentiated and well/moderately differentiated groups with the independent samples *t* test or Mann-Whitney U test, and categorical variables were compared with the chi-square test or Fisher’s exact test. Kolmogorov Smirnov test was used to test the normality of the continuous variables. Logistic regression was used to assess the association between the degree of differentiation on histopathology specimens and imaging parameters by APTw and mDixon-Quant. The receiver operator characteristic (ROC) curve was used to evaluate the diagnostic efficacy of different parameters. From the ROC curves, the area under the curve (AUC), threshold, sensitivity and specificity were obtained. The difference in AUCs between models was detected with the Delong test using MedCalcv15.2.2 software (MedCalc Software, Ostend, Belgium). The Pearson correlation coefficient was used to evaluate correlations between the APTw and mDixon-Quant parameters. A P-value < 0.05 was considered statistically significant.

## 3 Results

### 3.1 Comparison of the clinical data between the poorly differentiated and well/moderately differentiated groups

Age, menopause status, and vaginal bleeding of the patients in the poorly differentiated and well/moderately differentiated groups are persented in **Table 1**. There was no significant difference in clinical indices between the two groups (P > 0.05).

### 3.2 Data consistency between the two observers

The intra-class agreements between the two observers were good for the APTw, FF, and R2* values in both groups (all ICC ≥ 0.75, **Table 3**).

**Table 3:** APTw, FF, and ${\text{R}}_{2}^{*}$ measurement consistency between two observers.

		Observer 1	Observer 2	ICC
APTw (%)	poorly differentiated group	2.95 ± 0.79	2.94 ± 0.80	0.959
	well/moderately differentiated group	2.10(1.80, 2.70)	2.20(1.70, 2.40)	0.963
FF (%)	poorly differentiated group	2.17(1.17, 3.38)	2.32 ± 1.20	0.899
	well/moderately differentiated group	2.42 ± 0.84	2.32 ± 0.88	0.813
R2* (/s)	poorly differentiated group	28.09 ± 7.90	27.25 ± 6.99	0.921
	well/moderately differentiated group	22.13 ± 5.81	23.72 ± 6.73	0.936

ICC, intraclass correlation coeifficient; APTw, amide proton transfer weighted imaging; FF, fat fraction.

### 3.3 Comparison of parameters between the two groups

The APTw and R2* values of the poorly differentiated group were significantly higher than those of the well/moderately differentiated group (P < 0.05, **Table 4**). There were no statistically significant difference in FF measurements between the two groups (P > 0.05). Representative images from a patient in the poorly differentiated group and one in the well/moderately differentiated group are presented in **Figure 1**.

**Table 4:** Comparison of APTw and mDixon-Quant parameters between the two groups.

	poorly differentiated group (n=37)	well/moderately differentiated group (n=15)	z values	P value
APTw (%)	2.95 ± 0.78	2.05 (1.85, 2.65)	-2.940	0.003
FF (%)	2.13 (1.32, 3.09)	2.37 ± 0.79	-0.495	0.621
R2* (/s)	26.62 (21.99, 33.31)	22.93 ± 6.09	-2.090	0.037

APTw, amide proton transfer weighted imaging; FF, fat fraction.

### 3.4 Diagnostic performance of each parameter and their combination for diagnostic efficacy

The APTw and R2* values with significant difference between the two histopathologic groups were included in the ROC test (**Table 5**). The AUCs of APTw and R2* values for diagnosis of poorly and well/moderately differenciation of CSC were 0.762 and 0.686, respectively. The combined R2* value signifcantly improve the diagnostic performance (AUC = 0.843). The result of the Delong test suggested a statistical significance between AUCs by R2* and combined APTw + R2* values (**Table 6**).

**Table 5:** Diagnostic efficacy of each parameter.

	AUC	Sensitivity (%)	Specificity (%)	Threshold value
APTw	0.762	81.1	73.3	2.25%
R2*	0.686	51.4	80	26.53(/s)
$\text{APTw}+{\text{R}}_{2}^{*}$	0.843	67.6	93.3	—

AUC, area under the curve; APTw, amide proton transfer weighted imaging; APTw + R2* , APTw combined with R2*.

**Table 6:** Receiver operating characteristic curve paired with the Delong test.

	Z-values	P-values
APTw vs. R2*	0.629	0.5294
APTw vs. APTw + R2*	1.189	0.2345
R2* vs. APTw + R2*	2.284	0.0224

APTw, amide proton transfer weighted imaging; APTw + R2* , APTw combined with R2*.

R2* values were significantly correlated with APTw values in the poorly differentiated group (P<0.05) (**Table 7**).

**Table 7:** Correlations between the APTw and mDixon-Quant parameters.

	Poorly differentiated group		Well/moderately differentiated group	
APTw			APTw	
	r values	P-values	r values	P-values
FF	0.089	0.599	0.074	0.793
R_2_^*^	-0.394	0.016	-0.188	0.503

FF, fat fraction; APTw, amide proton transfer weighted imaging.

## 4 Discussion

APTw and mDixon-Quant imaging were evaluated in this study for discrimination of the differention degree of CSC by histopathology. The APTw and R2* values of the poorly differentiated group were significantly higher than those of the well/moderately differentiated group. And the combination of APTw and R2* values showed a high diagnostic efficacy in discrimination of CSC with different differention degrees.

A previous study on cervical cancer showed that moderately and poorly differentiated tumors were more common than well-differentiated tumors (22). In this study, the sample size of the poorly differentiated group was also much larger than that of the well/moderately differentiated group. The treatment scheme of CSC is highly based on the degree of histopathologic differentiation. Compared with well-differentiated CSC, poorly differentiated CSC has a poorer prognosis and shorter survival time. In a study by McCluggage et al, the total survival time of patients with well, moderately, and poorly differentiated CSC was 143.4 months, 124.2 months, and 86.1 months, respectively (23).

Early cervical cancer is primarily asymptomatic and may be accompanied by watery vaginal secretions, post-coital bleeding, or intermittent punctate bleeding. Usually, these early symptoms are not noticed by patients, and the disease often becomes more serious with delays in treatment. In our study, older postmenopausal patients with abnormal vaginal bleeding were common; however, the clinical manifestations of patients in the poorly differentiated and well/moderately differentiated CSC groups were similar.

The R2* value can be non-invasively determined by the mDixon Quant with elimination of the interference from fat signals. İdilman et al. (24) evaluated iron concentrations with ${\text{R}}_{2}^{*}$ values in fat-rich organs, such as the liver, pancreas, and bone marrow, and found that the iron content in organs was underestimated without fat suppression and significantly improved after fat suppression. In this study, the R2* values of poorly differentiated tumors were significantly higher than those of the well/moderately differentiated tumors. As we know, R2* is sensitive to local magnetic field inhomogeneity (25). When the paramagnetic material of blood metabolites including deoxyhemoglobin and hemosiderin produce local inhomogeneous magnetic field, the R2* value increases (26). In cervical cancer, the growth and metabolism in poorly differentiated tumor cells can be more vigorous and tissue oxygen consumption increases, resulting in a state of hypoxia and a need for more nutrients, such as sugar, protein, and oxygen (27). An increase in the concentration of paramagnetic material ensues with an increase in the R2* value.

The results of this study showed that the APTw values of poorly differentiated CSC were significantly higher than those of well/moderately differentiated CSC. Liu et al. showed that tumor cell densities and tumor pathologic grades positively correlated in patients with uterine cervical cancer (28). In our study, the poorly differentiated CSC were commonly with a higher degree of malignancy compared with well/moderately differentiated CSC. Therefore, poorly differentiated CSC cells should have more active proliferation and higher cell densities than well/moderately differentiated CSC cells, allowing additional free proteins and polypeptides to be synthesized. APTw signal reflects free protein and polypeptide concentrations in tissues, and can be affected by the heterogeneity and composition of tumors (16). Thus, APTw values in poorly differentiated cancer types are higher than those in well/moderately differentiated cancer types, as has been reported previously (29–31).

Combination of APTw and R2* image analysis can provide information about cell proliferation by measuring the changes in protein concentrations and iron content in tumor microenvironments, and thus showed improved diagnosis between poorly and well/moderately CSC. APTw and R2* values were significantly correlated in poorly differentiated CSC cases but not in the well/moderately differentiated CSC cases. These observations need verification with research on larger samples.

This study has some limitations. First, the sample size is relatively small, and thus the well- and moderately- differentiated CSC were combined into one group. As a result, the difference between the two degrees of differentiation were not fully compared. Also limited by sample size, microsatellite instability and other immunohistochemical indexes have not been studied in detail. Further studies with increased sample sizes are expected to exploit the capacity of APTw to differentiate other prognostic factors and predict cervical cancer prognosis.

## 5 Conclusions

APTw and mDixon-Quant imaging were investegated for clinical non-invasive evaluation of CSC differentiation. APTw combined with ${\text{R}}_{2}^{*}$ values showed a high efficacy in discriminating poorly from well/moderately differentiated CSC, and may help the treatment design and prognosis prediction of CSC.

## Data availability statement

The raw data supporting the conclusions of this article will be made available by the authors, without undue reservation.

## Ethics statement

The studies involving human participants were reviewed and approved by The First Affiliated Hospital of Dalian Medical University. Written informed consent for participation was not required for this study in accordance with the national legislation and the institutional requirements.

## Author contributions

XM and AL conceived of the presented idea. XM and CM performed the measurements. ST were supervised the work. XM and LL processed the experimental data, performed the analysis. XM drafted the manuscript, and LL, XZ and JW aided in working on the manuscript. QS carries out image scanning. All authors discussed the results and contributed to the final manuscript. All authors contributed to the article and approved the submitted version.
